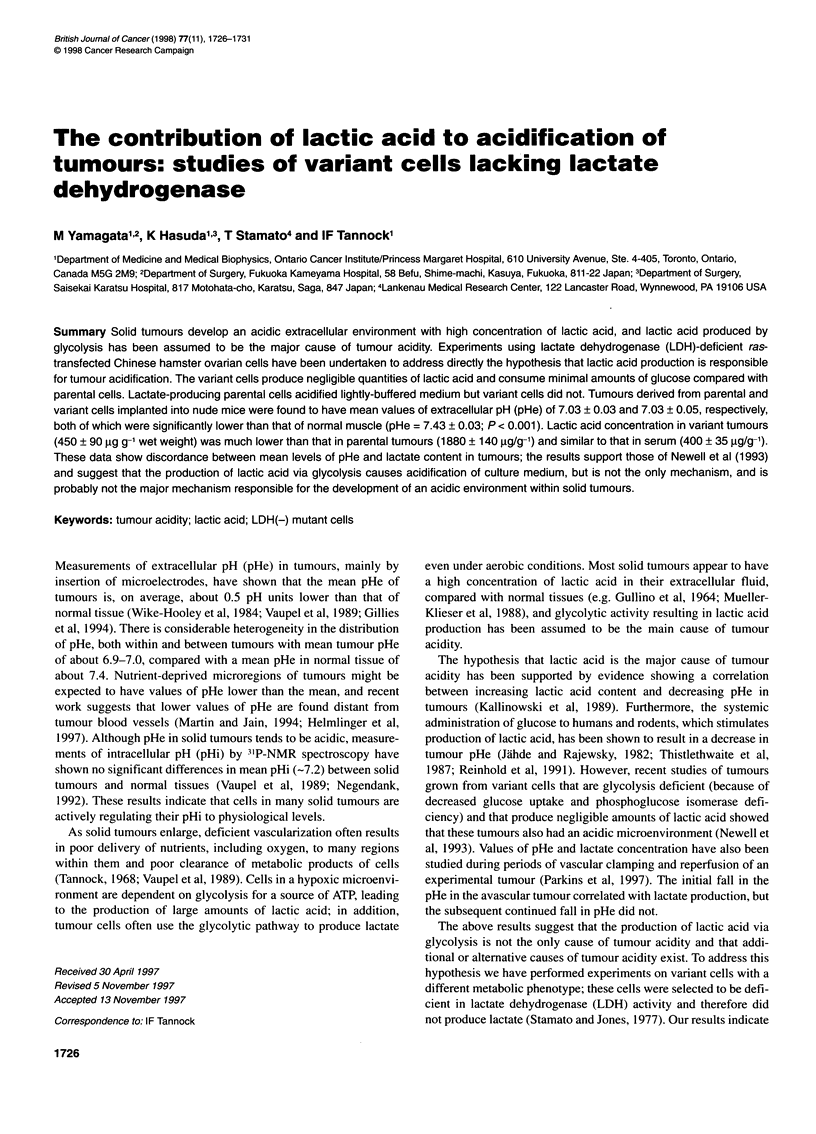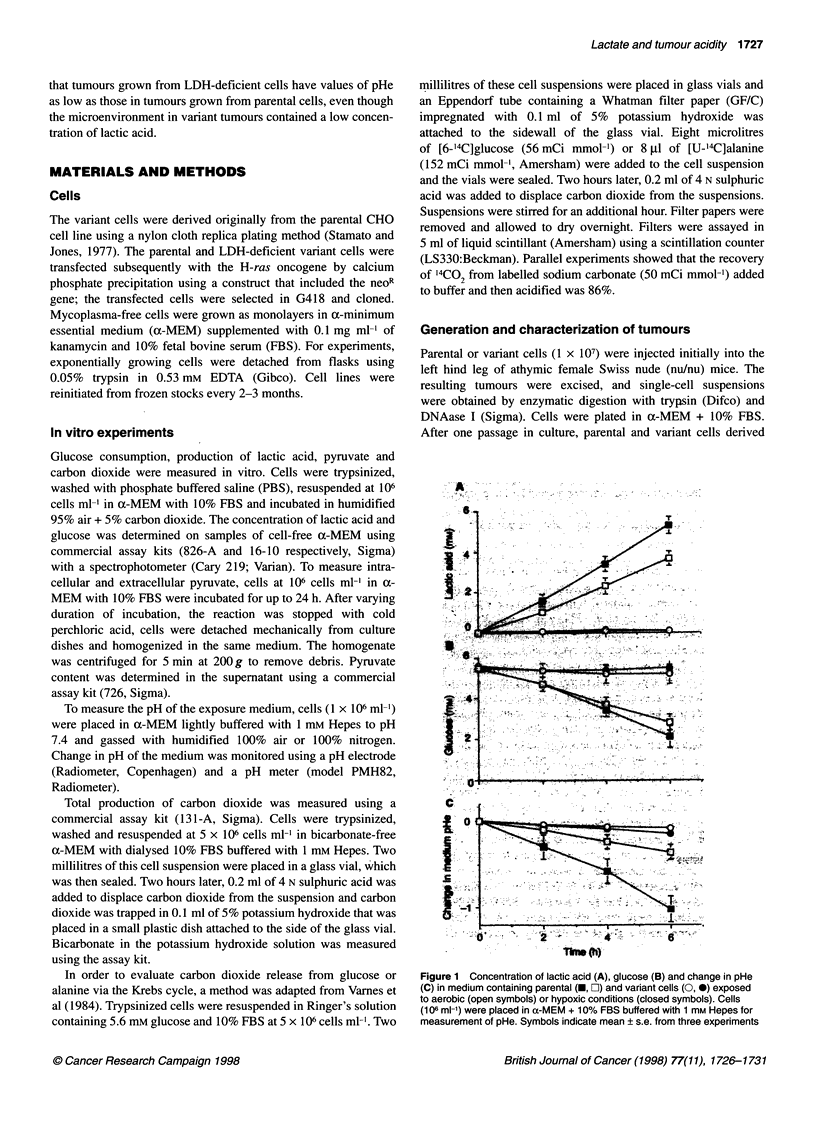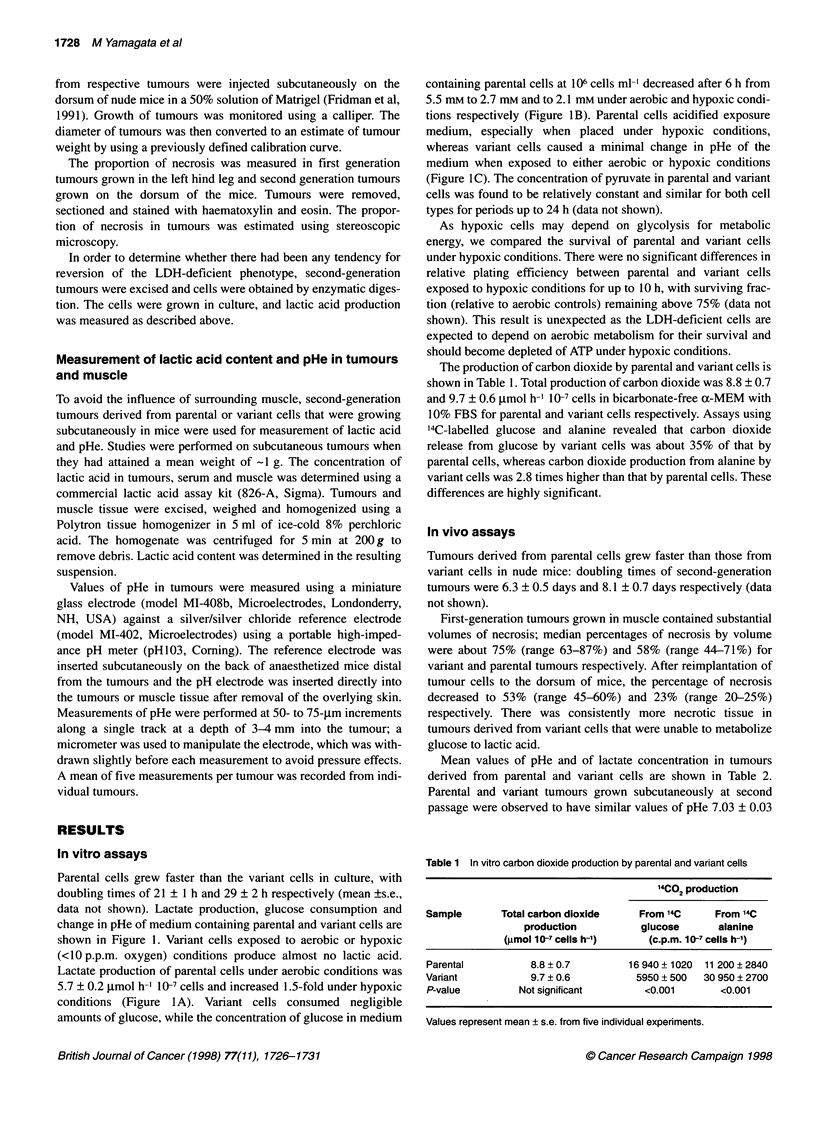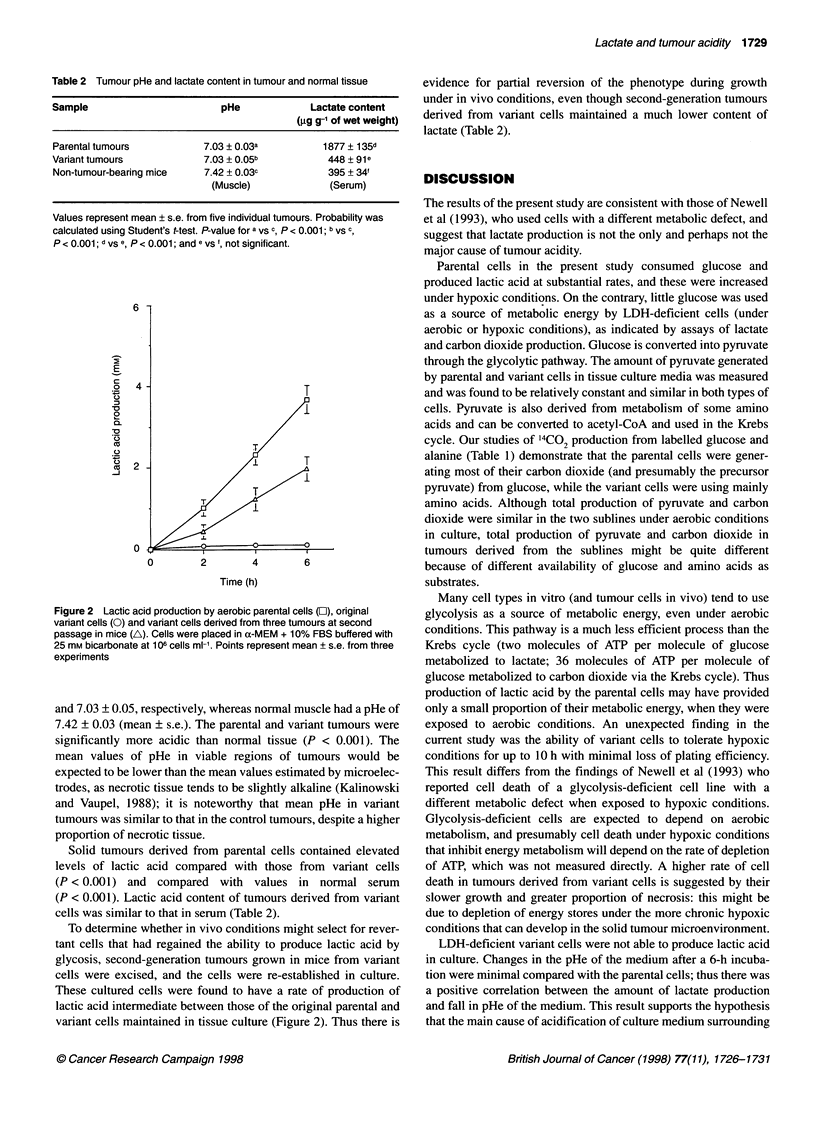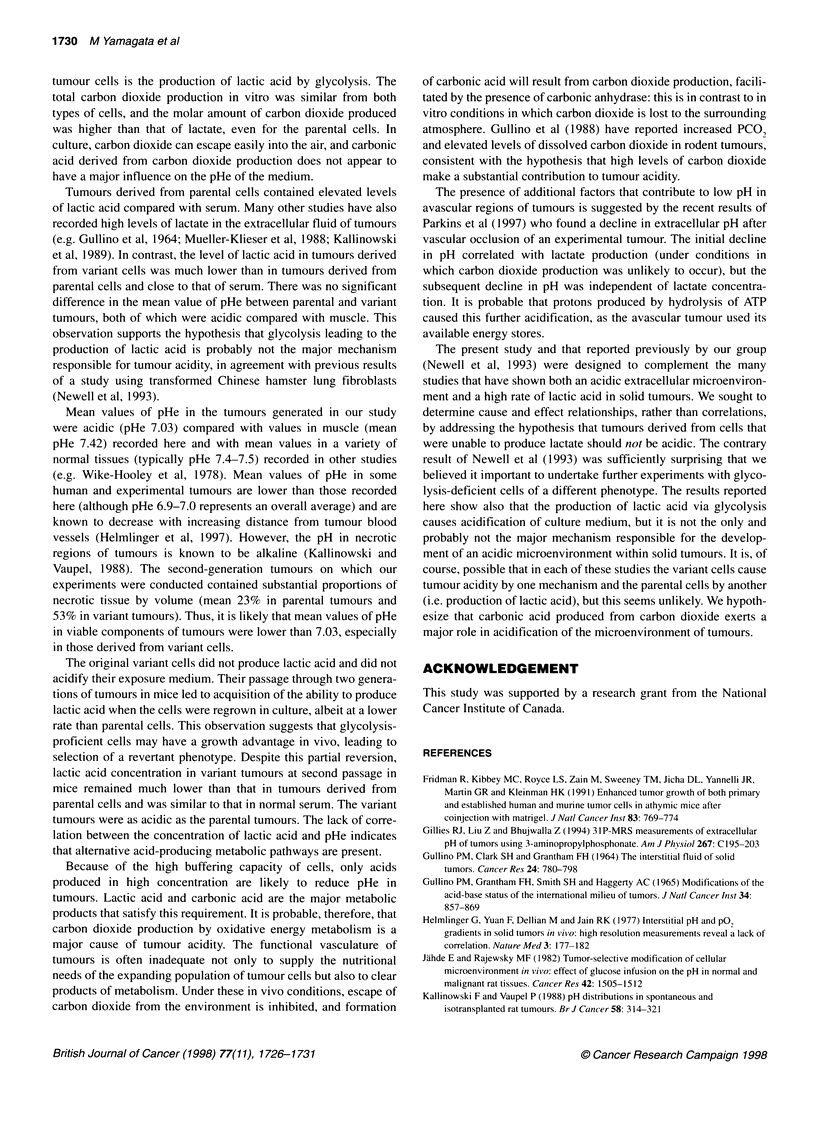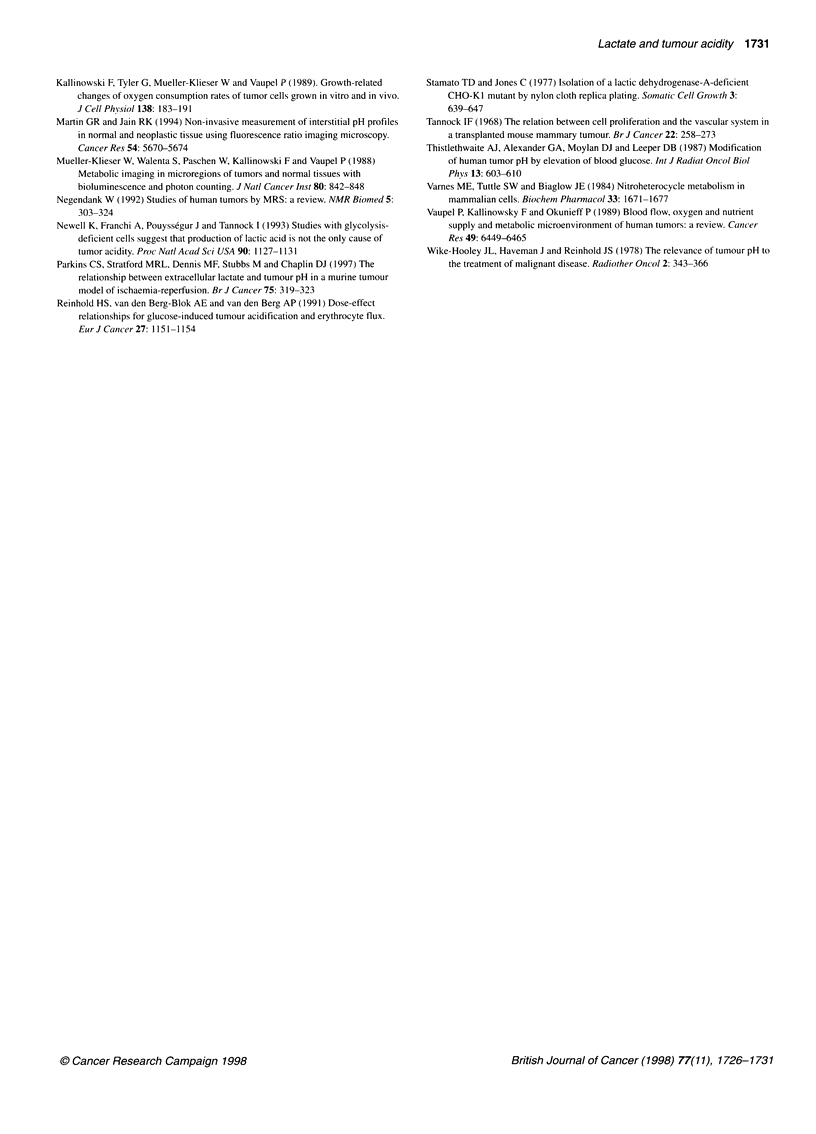# The contribution of lactic acid to acidification of tumours: studies of variant cells lacking lactate dehydrogenase.

**DOI:** 10.1038/bjc.1998.289

**Published:** 1998-06

**Authors:** M. Yamagata, K. Hasuda, T. Stamato, I. F. Tannock

**Affiliations:** Department of Medicine and Medical Biophysics, Ontario Cancer Institute/Princess Margaret Hospital, Toronto, Canada.

## Abstract

Solid tumours develop an acidic extracellular environment with high concentration of lactic acid, and lactic acid produced by glycolysis has been assumed to be the major cause of tumour acidity. Experiments using lactate dehydrogenase (LDH)-deficient ras-transfected Chinese hamster ovarian cells have been undertaken to address directly the hypothesis that lactic acid production is responsible for tumour acidification. The variant cells produce negligible quantities of lactic acid and consume minimal amounts of glucose compared with parental cells. Lactate-producing parental cells acidified lightly-buffered medium but variant cells did not. Tumours derived from parental and variant cells implanted into nude mice were found to have mean values of extracellular pH (pHe) of 7.03 +/- 0.03 and 7.03 +/- 0.05, respectively, both of which were significantly lower than that of normal muscle (pHe = 7.43 +/- 0.03; P < 0.001). Lactic acid concentration in variant tumours (450 +/- 90 microg g(-1) wet weight) was much lower than that in parental tumours (1880 +/- 140 microg/g(-1)) and similar to that in serum (400 +/- 35 microg/g(-1)). These data show discordance between mean levels of pHe and lactate content in tumours; the results support those of Newell et al (1993) and suggest that the production of lactic acid via glycolysis causes acidification of culture medium, but is not the only mechanism, and is probably not the major mechanism responsible for the development of an acidic environment within solid tumours.


British Journal of Cancer (1998) 77(11), 1726–1731
© 1998 Cancer Research Campaign

### The contribution of lactic acid to acidification of tumours: studies of variant cells lacking lactate dehydrogenase

**M Yamagata[1,2], K Hasuda[1,3], T Stamato[4] and IF Tannock[1]**

[1]Department of Medicine and Medical Biophysics, Ontario Cancer Institute/Princess Margaret Hospital, 610 University Avenue, Ste. 4-405, Toronto, Ontario, Canada M5G 2M9; [2]Department of Surgery, Fukuoka Kameyama Hospital, 58 Befu, Shime-machi, Kasuya, Fukuoka, 811-22 Japan; [3]Department of Surgery, Saisekai Karatsu Hospital, 817 Motohata-cho, Karatsu, Saga, 847 Japan; [4]Lankenau Medical Research Center, 122 Lancaster Road, Wynnewood, PA 19106 USA

**Summary** Solid tumours develop an acidic extracellular environment with high concentration of lactic acid, and lactic acid produced by glycolysis has been assumed to be the major cause of tumour acidity. Experiments using lactate dehydrogenase (LDH)-deficient *ras*-transfected Chinese hamster ovarian cells have been undertaken to address directly the hypothesis that lactic acid production is responsible for tumour acidification. The variant cells produce negligible quantities of lactic acid and consume minimal amounts of glucose compared with parental cells. Lactate-producing parental cells acidified lightly-buffered medium but variant cells did not. Tumours derived from parental and variant cells implanted into nude mice were found to have mean values of extracellular pH (pHe) of 7.03 ± 0.03 and 7.03 ± 0.05, respectively, both of which were significantly lower than that of normal muscle (pHe = 7.43 ± 0.03; $P < 0.001$). Lactic acid concentration in variant tumours (450 ± 90 μg $g^{-1}$ wet weight) was much lower than that in parental tumours (1880 ± 140 μg/$g^{-1}$) and similar to that in serum (400 ± 35 μg/$g^{-1}$). These data show discordance between mean levels of pHe and lactate content in tumours; the results support those of Newell et al (1993) and suggest that the production of lactic acid via glycolysis causes acidification of culture medium, but is not the only mechanism, and is probably not the major mechanism responsible for the development of an acidic environment within solid tumours.

**Keywords:** tumour acidity; lactic acid; LDH(–) mutant cells

Measurements of extracellular pH (pHe) in tumours, mainly by insertion of microelectrodes, have shown that the mean pHe of tumours is, on average, about 0.5 pH units lower than that of normal tissue (Wike-Hooley et al, 1984; Vaupel et al, 1989; Gillies et al, 1994). There is considerable heterogeneity in the distribution of pHe, both within and between tumours with mean tumour pHe of about 6.9–7.0, compared with a mean pHe in normal tissue of about 7.4. Nutrient-deprived microregions of tumours might be expected to have values of pHe lower than the mean, and recent work suggests that lower values of pHe are found distant from tumour blood vessels (Martin and Jain, 1994; Helmlinger et al, 1997). Although pHe in solid tumours tends to be acidic, measurements of intracellular pH (pHi) by $^{31}$P-NMR spectroscopy have shown no significant differences in mean pHi (~7.2) between solid tumours and normal tissues (Vaupel et al, 1989; Negendank, 1992). These results indicate that cells in many solid tumours are actively regulating their pHi to physiological levels.

As solid tumours enlarge, deficient vascularization often results in poor delivery of nutrients, including oxygen, to many regions within them and poor clearance of metabolic products of cells (Tannock, 1968; Vaupel et al, 1989). Cells in a hypoxic microenvironment are dependent on glycolysis for a source of ATP, leading to the production of large amounts of lactic acid; in addition, tumour cells often use the glycolytic pathway to produce lactate even under aerobic conditions. Most solid tumours appear to have a high concentration of lactic acid in their extracellular fluid, compared with normal tissues (e.g. Gullino et al, 1964; Mueller-Klieser et al, 1988), and glycolytic activity resulting in lactic acid production has been assumed to be the main cause of tumour acidity.

The hypothesis that lactic acid is the major cause of tumour acidity has been supported by evidence showing a correlation between increasing lactic acid content and decreasing pHe in tumours (Kallinowski et al, 1989). Furthermore, the systemic administration of glucose to humans and rodents, which stimulates production of lactic acid, has been shown to result in a decrease in tumour pHe (Jähde and Rajewsky, 1982; Thistlethwaite et al, 1987; Reinhold et al, 1991). However, recent studies of tumours grown from variant cells that are glycolysis deficient (because of decreased glucose uptake and phosphoglucose isomerase deficiency) and that produce negligible amounts of lactic acid showed that these tumours also had an acidic microenvironment (Newell et al, 1993). Values of pHe and lactate concentration have also been studied during periods of vascular clamping and reperfusion of an experimental tumour (Parkins et al, 1997). The initial fall in the pHe in the avascular tumour correlated with lactate production, but the subsequent continued fall in pHe did not.

The above results suggest that the production of lactic acid via glycolysis is not the only cause of tumour acidity and that additional or alternative causes of tumour acidity exist. To address this hypothesis we have performed experiments on variant cells with a different metabolic phenotype; these cells were selected to be deficient in lactate dehydrogenase (LDH) activity and therefore did not produce lactate (Stamato and Jones, 1977). Our results indicate

*Received 30 April 1997*
*Revised 5 November 1997*
*Accepted 13 November 1997*

*Correspondence to:* IF Tannock that tumours grown from LDH-deficient cells have values of pHe as low as those in tumours grown from parental cells, even though the microenvironment in variant tumours contained a low concentration of lactic acid.

### MATERIALS AND METHODS

### Cells

The variant cells were derived originally from the parental CHO cell line using a nylon cloth replica plating method (Stamato and Jones, 1977). The parental and LDH-deficient variant cells were transfected subsequently with the H-*ras* oncogene by calcium phosphate precipitation using a construct that included the neo$^R$ gene; the transfected cells were selected in G418 and cloned. Mycoplasma-free cells were grown as monolayers in α-minimum essential medium (α-MEM) supplemented with 0.1 mg $ml^{-1}$ of kanamycin and 10% fetal bovine serum (FBS). For experiments, exponentially growing cells were detached from flasks using 0.05% trypsin in 0.53 mM EDTA (Gibco). Cell lines were reinitiated from frozen stocks every 2–3 months.

### In vitro experiments

Glucose consumption, production of lactic acid, pyruvate and carbon dioxide were measured in vitro. Cells were trypsinized, washed with phosphate buffered saline (PBS), resuspended at $10^6$ cells $ml^{-1}$ in α-MEM with 10% FBS and incubated in humidified 95% air + 5% carbon dioxide. The concentration of lactic acid and glucose was determined on samples of cell-free α-MEM using commercial assay kits (826-A and 16-10 respectively, Sigma) with a spectrophotometer (Cary 219; Varian). To measure intracellular and extracellular pyruvate, cells at $10^6$ cells $ml^{-1}$ in α-MEM with 10% FBS were incubated for up to 24 h. After varying duration of incubation, the reaction was stopped with cold perchloric acid, cells were detached mechanically from culture dishes and homogenized in the same medium. The homogenate was centrifuged for 5 min at 200 ***g*** to remove debris. Pyruvate content was determined in the supernatant using a commercial assay kit (726, Sigma).

To measure the pH of the exposure medium, cells ($1 \times 10^6$ $ml^{-1}$) were placed in α-MEM lightly buffered with 1 mM Hepes to pH 7.4 and gassed with humidified 100% air or 100% nitrogen. Change in pH of the medium was monitored using a pH electrode (Radiometer, Copenhagen) and a pH meter (model PMH82, Radiometer).

Total production of carbon dioxide was measured using a commercial assay kit (131-A, Sigma). Cells were trypsinized, washed and resuspended at $5 \times 10^6$ cells $ml^{-1}$ in bicarbonate-free α-MEM with dialysed 10% FBS buffered with 1 mM Hepes. Two millilitres of this cell suspension were placed in a glass vial, which was then sealed. Two hours later, 0.2 ml of 4 N sulphuric acid was added to displace carbon dioxide from the suspension and carbon dioxide was trapped in 0.1 ml of 5% potassium hydroxide that was placed in a small plastic dish attached to the side of the glass vial. Bicarbonate in the potassium hydroxide solution was measured using the assay kit.

In order to evaluate carbon dioxide release from glucose or alanine via the Krebs cycle, a method was adapted from Varnes et al (1984). Trypsinized cells were resuspended in Ringer's solution containing 5.6 mM glucose and 10% FBS at $5 \times 10^6$ cells $ml^{-1}$. Two millilitres of these cell suspensions were placed in glass vials and an Eppendorf tube containing a Whatman filter paper (GF/C) impregnated with 0.1 ml of 5% potassium hydroxide was attached to the sidewall of the glass vial. Eight microlitres of [6-$^{14}$C]glucose (56 mCi $mmol^{-1}$) or 8 µl of [U-$^{14}$C]alanine (152 mCi $mmol^{-1}$, Amersham) were added to the cell suspension and the vials were sealed. Two hours later, 0.2 ml of 4 N sulphuric acid was added to displace carbon dioxide from the suspensions. Suspensions were stirred for an additional hour. Filter papers were removed and allowed to dry overnight. Filters were assayed in 5 ml of liquid scintillant (Amersham) using a scintillation counter (LS330:Beckman). Parallel experiments showed that the recovery of $^{14}CO_2$ from labelled sodium carbonate (50 mCi $mmol^{-1}$) added to buffer and then acidified was 86%.

### Generation and characterization of tumours

Parental or variant cells ($1 \times 10^7$) were injected initially into the left hind leg of athymic female Swiss nude (nu/nu) mice. The resulting tumours were excised, and single-cell suspensions were obtained by enzymatic digestion with trypsin (Difco) and DNAase I (Sigma). Cells were plated in α-MEM + 10% FBS. After one passage in culture, parental and variant cells derived

**Figure 1** Concentration of lactic acid (**A**), glucose (**B**) and change in pHe (**C**) in medium containing parental (■, □) and variant cells (○, ●) exposed to aerobic (open symbols) or hypoxic conditions (closed symbols). Cells ($10^6$ $ml^{-1}$) were placed in α-MEM + 10% FBS buffered with 1 mM Hepes for measurement of pHe. Symbols indicate mean ± s.e. from three experiments

© Cancer Research Campaign 1998

from respective tumours were injected subcutaneously on the dorsum of nude mice in a 50% solution of Matrigel (Fridman et al, 1991). Growth of tumours was monitored using a calliper. The diameter of tumours was then converted to an estimate of tumour weight by using a previously defined calibration curve.

The proportion of necrosis was measured in first generation tumours grown in the left hind leg and second generation tumours grown on the dorsum of the mice. Tumours were removed, sectioned and stained with haematoxylin and eosin. The proportion of necrosis in tumours was estimated using stereoscopic microscopy.

In order to determine whether there had been any tendency for reversion of the LDH-deficient phenotype, second-generation tumours were excised and cells were obtained by enzymatic digestion. The cells were grown in culture, and lactic acid production was measured as described above.

### Measurement of lactic acid content and pHe in tumours and muscle

To avoid the influence of surrounding muscle, second-generation tumours derived from parental or variant cells that were growing subcutaneously in mice were used for measurement of lactic acid and pHe. Studies were performed on subcutaneous tumours when they had attained a mean weight of ~1 g. The concentration of lactic acid in tumours, serum and muscle was determined using a commercial lactic acid assay kit (826-A, Sigma). Tumours and muscle tissue were excised, weighed and homogenized using a Polytron tissue homogenizer in 5 ml of ice-cold 8% perchloric acid. The homogenate was centrifuged for 5 min at 200 ***g*** to remove debris. Lactic acid content was determined in the resulting suspension.

Values of pHe in tumours were measured using a miniature glass electrode (model MI-408b, Microelectrodes, Londonderry, NH, USA) against a silver/silver chloride reference electrode (model MI-402, Microelectrodes) using a portable high-impedance pH meter (pH103, Corning). The reference electrode was inserted subcutaneously on the back of anaesthetized mice distal from the tumours and the pH electrode was inserted directly into the tumours or muscle tissue after removal of the overlying skin. Measurements of pHe were performed at 50- to 75-μm increments along a single track at a depth of 3–4 mm into the tumour; a micrometer was used to manipulate the electrode, which was withdrawn slightly before each measurement to avoid pressure effects. A mean of five measurements per tumour was recorded from individual tumours.

### RESULTS

### In vitro assays

Parental cells grew faster than the variant cells in culture, with doubling times of 21 ± 1 h and 29 ± 2 h respectively (mean ±s.e., data not shown). Lactate production, glucose consumption and change in pHe of medium containing parental and variant cells are shown in Figure 1. Variant cells exposed to aerobic or hypoxic (<10 p.p.m. oxygen) conditions produce almost no lactic acid. Lactate production of parental cells under aerobic conditions was 5.7 ± 0.2 μmol $h^{-1}$ $10^{-7}$ cells and increased 1.5-fold under hypoxic conditions (Figure 1A). Variant cells consumed negligible amounts of glucose, while the concentration of glucose in medium containing parental cells at $10^6$ cells $ml^{-1}$ decreased after 6 h from 5.5 mM to 2.7 mM and to 2.1 mM under aerobic and hypoxic conditions respectively (Figure 1B). Parental cells acidified exposure medium, especially when placed under hypoxic conditions, whereas variant cells caused a minimal change in pHe of the medium when exposed to either aerobic or hypoxic conditions (Figure 1C). The concentration of pyruvate in parental and variant cells was found to be relatively constant and similar for both cell types for periods up to 24 h (data not shown).

As hypoxic cells may depend on glycolysis for metabolic energy, we compared the survival of parental and variant cells under hypoxic conditions. There were no significant differences in relative plating efficiency between parental and variant cells exposed to hypoxic conditions for up to 10 h, with surviving fraction (relative to aerobic controls) remaining above 75% (data not shown). This result is unexpected as the LDH-deficient cells are expected to depend on aerobic metabolism for their survival and should become depleted of ATP under hypoxic conditions.

The production of carbon dioxide by parental and variant cells is shown in Table 1. Total production of carbon dioxide was 8.8 ± 0.7 and 9.7 ± 0.6 μmol $h^{-1}$ $10^{-7}$ cells in bicarbonate-free α-MEM with 10% FBS for parental and variant cells respectively. Assays using $^{14}C$-labelled glucose and alanine revealed that carbon dioxide release from glucose by variant cells was about 35% of that by parental cells, whereas carbon dioxide production from alanine by variant cells was 2.8 times higher than that by parental cells. These differences are highly significant.

### In vivo assays

Tumours derived from parental cells grew faster than those from variant cells in nude mice: doubling times of second-generation tumours were 6.3 ± 0.5 days and 8.1 ± 0.7 days respectively (data not shown).

First-generation tumours grown in muscle contained substantial volumes of necrosis; median percentages of necrosis by volume were about 75% (range 63–87%) and 58% (range 44–71%) for variant and parental tumours respectively. After reimplantation of tumour cells to the dorsum of mice, the percentage of necrosis decreased to 53% (range 45–60%) and 23% (range 20–25%) respectively. There was consistently more necrotic tissue in tumours derived from variant cells that were unable to metabolize glucose to lactic acid.

Mean values of pHe and of lactate concentration in tumours derived from parental and variant cells are shown in Table 2. Parental and variant tumours grown subcutaneously at second passage were observed to have similar values of pHe 7.03 ± 0.03

**Table 1** In vitro carbon dioxide production by parental and variant cells

| Sample | Total carbon dioxide production (μmol $10^{-7}$ cells $h^{-1}$) | $^{14}CO_2$ production: From $^{14}C$ glucose (c.p.m. $10^{-7}$ cells $h^{-1}$) | $^{14}CO_2$ production: From $^{14}C$ alanine (c.p.m. $10^{-7}$ cells $h^{-1}$) |
|---|---|---|---|
| Parental | 8.8 ± 0.7 | 16 940 ± 1020 | 11 200 ± 2840 |
| Variant | 9.7 ± 0.6 | 5950 ± 500 | 30 950 ± 2700 |
| *P*-value | Not significant | <0.001 | <0.001 |

Values represent mean ± s.e. from five individual experiments.

© Cancer Research Campaign 1998

**Table 2** Tumour pHe and lactate content in tumour and normal tissue

| Sample | pHe | Lactate content (μg g$^{-1}$ of wet weight) |
|---|---|---|
| Parental tumours | 7.03 ± 0.03[a] | 1877 ± 135[d] |
| Variant tumours | 7.03 ± 0.05[b] | 448 ± 91[e] |
| Non-tumour-bearing mice | 7.42 ± 0.03[c] (Muscle) | 395 ± 34[f] (Serum) |

Values represent mean ± s.e. from five individual tumours. Probability was calculated using Student's *t*-test. *P*-value for [a] vs [c], $P < 0.001$; [b] vs [c], $P < 0.001$; [d] vs [e], $P < 0.001$; and [e] vs [f], not significant.

**Figure 2** Lactic acid production by aerobic parental cells (□), original variant cells (○) and variant cells derived from three tumours at second passage in mice (△). Cells were placed in α-MEM + 10% FBS buffered with 25 mM bicarbonate at $10^6$ cells ml$^{-1}$. Points represent mean ± s.e. from three experiments and 7.03 ± 0.05, respectively, whereas normal muscle had a pHe of 7.42 ± 0.03 (mean ± s.e.). The parental and variant tumours were significantly more acidic than normal tissue ($P < 0.001$). The mean values of pHe in viable regions of tumours would be expected to be lower than the mean values estimated by microelectrodes, as necrotic tissue tends to be slightly alkaline (Kalinowski and Vaupel, 1988); it is noteworthy that mean pHe in variant tumours was similar to that in the control tumours, despite a higher proportion of necrotic tissue.

Solid tumours derived from parental cells contained elevated levels of lactic acid compared with those from variant cells ($P < 0.001$) and compared with values in normal serum ($P < 0.001$). Lactic acid content of tumours derived from variant cells was similar to that in serum (Table 2).

To determine whether in vivo conditions might select for revertant cells that had regained the ability to produce lactic acid by glycosis, second-generation tumours grown in mice from variant cells were excised, and the cells were re-established in culture. These cultured cells were found to have a rate of production of lactic acid intermediate between those of the original parental and variant cells maintained in tissue culture (Figure 2). Thus there is evidence for partial reversion of the phenotype during growth under in vivo conditions, even though second-generation tumours derived from variant cells maintained a much lower content of lactate (Table 2).

### DISCUSSION

The results of the present study are consistent with those of Newell et al (1993), who used cells with a different metabolic defect, and suggest that lactate production is not the only and perhaps not the major cause of tumour acidity.

Parental cells in the present study consumed glucose and produced lactic acid at substantial rates, and these were increased under hypoxic conditions. On the contrary, little glucose was used as a source of metabolic energy by LDH-deficient cells (under aerobic or hypoxic conditions), as indicated by assays of lactate and carbon dioxide production. Glucose is converted into pyruvate through the glycolytic pathway. The amount of pyruvate generated by parental and variant cells in tissue culture media was measured and was found to be relatively constant and similar in both types of cells. Pyruvate is also derived from metabolism of some amino acids and can be converted to acetyl-CoA and used in the Krebs cycle. Our studies of $^{14}CO_2$ production from labelled glucose and alanine (Table 1) demonstrate that the parental cells were generating most of their carbon dioxide (and presumably the precursor pyruvate) from glucose, while the variant cells were using mainly amino acids. Although total production of pyruvate and carbon dioxide were similar in the two sublines under aerobic conditions in culture, total production of pyruvate and carbon dioxide in tumours derived from the sublines might be quite different because of different availability of glucose and amino acids as substrates.

Many cell types in vitro (and tumour cells in vivo) tend to use glycolysis as a source of metabolic energy, even under aerobic conditions. This pathway is a much less efficient process than the Krebs cycle (two molecules of ATP per molecule of glucose metabolized to lactate; 36 molecules of ATP per molecule of glucose metabolized to carbon dioxide via the Krebs cycle). Thus production of lactic acid by the parental cells may have provided only a small proportion of their metabolic energy, when they were exposed to aerobic conditions. An unexpected finding in the current study was the ability of variant cells to tolerate hypoxic conditions for up to 10 h with minimal loss of plating efficiency. This result differs from the findings of Newell et al (1993) who reported cell death of a glycolysis-deficient cell line with a different metabolic defect when exposed to hypoxic conditions. Glycolysis-deficient cells are expected to depend on aerobic metabolism, and presumably cell death under hypoxic conditions that inhibit energy metabolism will depend on the rate of depletion of ATP, which was not measured directly. A higher rate of cell death in tumours derived from variant cells is suggested by their slower growth and greater proportion of necrosis: this might be due to depletion of energy stores under the more chronic hypoxic conditions that can develop in the solid tumour microenvironment.

LDH-deficient variant cells were not able to produce lactic acid in culture. Changes in the pHe of the medium after a 6-h incubation were minimal compared with the parental cells; thus there was a positive correlation between the amount of lactate production and fall in pHe of the medium. This result supports the hypothesis that the main cause of acidification of culture medium surrounding

© Cancer Research Campaign 1998

tumour cells is the production of lactic acid by glycolysis. The total carbon dioxide production in vitro was similar from both types of cells, and the molar amount of carbon dioxide produced was higher than that of lactate, even for the parental cells. In culture, carbon dioxide can escape easily into the air, and carbonic acid derived from carbon dioxide production does not appear to have a major influence on the pHe of the medium.

Tumours derived from parental cells contained elevated levels of lactic acid compared with serum. Many other studies have also recorded high levels of lactate in the extracellular fluid of tumours (e.g. Gullino et al, 1964; Mueller-Klieser et al, 1988; Kallinowski et al, 1989). In contrast, the level of lactic acid in tumours derived from variant cells was much lower than in tumours derived from parental cells and close to that of serum. There was no significant difference in the mean value of pHe between parental and variant tumours, both of which were acidic compared with muscle. This observation supports the hypothesis that glycolysis leading to the production of lactic acid is probably not the major mechanism responsible for tumour acidity, in agreement with previous results of a study using transformed Chinese hamster lung fibroblasts (Newell et al, 1993).

Mean values of pHe in the tumours generated in our study were acidic (pHe 7.03) compared with values in muscle (mean pHe 7.42) recorded here and with mean values in a variety of normal tissues (typically pHe 7.4–7.5) recorded in other studies (e.g. Wike-Hooley et al, 1978). Mean values of pHe in some human and experimental tumours are lower than those recorded here (although pHe 6.9–7.0 represents an overall average) and are known to decrease with increasing distance from tumour blood vessels (Helmlinger et al, 1997). However, the pH in necrotic regions of tumours is known to be alkaline (Kallinowski and Vaupel, 1988). The second-generation tumours on which our experiments were conducted contained substantial proportions of necrotic tissue by volume (mean 23% in parental tumours and 53% in variant tumours). Thus, it is likely that mean values of pHe in viable components of tumours were lower than 7.03, especially in those derived from variant cells.

The original variant cells did not produce lactic acid and did not acidify their exposure medium. Their passage through two generations of tumours in mice led to acquisition of the ability to produce lactic acid when the cells were regrown in culture, albeit at a lower rate than parental cells. This observation suggests that glycolysis-proficient cells may have a growth advantage in vivo, leading to selection of a revertant phenotype. Despite this partial reversion, lactic acid concentration in variant tumours at second passage in mice remained much lower than that in tumours derived from parental cells and was similar to that in normal serum. The variant tumours were as acidic as the parental tumours. The lack of correlation between the concentration of lactic acid and pHe indicates that alternative acid-producing metabolic pathways are present.

Because of the high buffering capacity of cells, only acids produced in high concentration are likely to reduce pHe in tumours. Lactic acid and carbonic acid are the major metabolic products that satisfy this requirement. It is probable, therefore, that carbon dioxide production by oxidative energy metabolism is a major cause of tumour acidity. The functional vasculature of tumours is often inadequate not only to supply the nutritional needs of the expanding population of tumour cells but also to clear products of metabolism. Under these in vivo conditions, escape of carbon dioxide from the environment is inhibited, and formation of carbonic acid will result from carbon dioxide production, facilitated by the presence of carbonic anhydrase: this is in contrast to in vitro conditions in which carbon dioxide is lost to the surrounding atmosphere. Gullino et al (1988) have reported increased $PCO_2$ and elevated levels of dissolved carbon dioxide in rodent tumours, consistent with the hypothesis that high levels of carbon dioxide make a substantial contribution to tumour acidity.

The presence of additional factors that contribute to low pH in avascular regions of tumours is suggested by the recent results of Parkins et al (1997) who found a decline in extracellular pH after vascular occlusion of an experimental tumour. The initial decline in pH correlated with lactate production (under conditions in which carbon dioxide production was unlikely to occur), but the subsequent decline in pH was independent of lactate concentration. It is probable that protons produced by hydrolysis of ATP caused this further acidification, as the avascular tumour used its available energy stores.

The present study and that reported previously by our group (Newell et al, 1993) were designed to complement the many studies that have shown both an acidic extracellular microenvironment and a high rate of lactic acid in solid tumours. We sought to determine cause and effect relationships, rather than correlations, by addressing the hypothesis that tumours derived from cells that were unable to produce lactate should *not* be acidic. The contrary result of Newell et al (1993) was sufficiently surprising that we believed it important to undertake further experiments with glycolysis-deficient cells of a different phenotype. The results reported here show also that the production of lactic acid via glycolysis causes acidification of culture medium, but it is not the only and probably not the major mechanism responsible for the development of an acidic microenvironment within solid tumours. It is, of course, possible that in each of these studies the variant cells cause tumour acidity by one mechanism and the parental cells by another (i.e. production of lactic acid), but this seems unlikely. We hypothesize that carbonic acid produced from carbon dioxide exerts a major role in acidification of the microenvironment of tumours.

### ACKNOWLEDGEMENT

This study was supported by a research grant from the National Cancer Institute of Canada.

### REFERENCES

Fridman R, Kibbey MC, Royce LS, Zain M, Sweeney TM, Jicha DL, Yannelli JR, Martin GR and Kleinman HK (1991) Enhanced tumor growth of both primary and established human and murine tumor cells in athymic mice after coinjection with matrigel. *J Natl Cancer Inst* **83**: 769–774

Gillies RJ, Liu Z and Bhujwalla Z (1994) 31P-MRS measurements of extracellular pH of tumors using 3-aminopropylphosphonate. *Am J Physiol* **267**: C195–203

Gullino PM, Clark SH and Grantham FH (1964) The interstitial fluid of solid tumors. *Cancer Res* **24**: 780–798

Gullino PM, Grantham FH, Smith SH and Haggerty AC (1965) Modifications of the acid-base status of the international milieu of tumors. *J Natl Cancer Inst* **34**: 857–869

Helmlinger G, Yuan F, Dellian M and Jain RK (1977) Interstitial pH and $pO_2$ gradients in solid tumors *in vivo*: high resolution measurements reveal a lack of correlation. *Nature Med* **3**: 177–182

Jähde E and Rajewsky MF (1982) Tumor-selective modification of cellular microenvironment *in vivo*: effect of glucose infusion on the pH in normal and malignant rat tissues. *Cancer Res* **42**: 1505–1512

Kallinowski F and Vaupel P (1988) pH distributions in spontaneous and isotransplanted rat tumours. *Br J Cancer* **58**: 314–321

© Cancer Research Campaign 1998

Kallinowski F, Tyler G, Mueller-Klieser W and Vaupel P (1989). Growth-related changes of oxygen consumption rates of tumor cells grown in vitro and in vivo. *J Cell Physiol* **138**: 183–191

Martin GR and Jain RK (1994) Non-invasive measurement of interstitial pH profiles in normal and neoplastic tissue using fluorescence ratio imaging microscopy. *Cancer Res* **54**: 5670–5674

Mueller-Klieser W, Walenta S, Paschen W, Kallinowski F and Vaupel P (1988) Metabolic imaging in microregions of tumors and normal tissues with bioluminescence and photon counting. *J Natl Cancer Inst* **80**: 842–848

Negendank W (1992) Studies of human tumors by MRS: a review. *NMR Biomed* **5**: 303–324

Newell K, Franchi A, Pouysségur J and Tannock I (1993) Studies with glycolysis-deficient cells suggest that production of lactic acid is not the only cause of tumor acidity. *Proc Natl Acad Sci USA* **90**: 1127–1131

Parkins CS, Stratford MRL, Dennis MF, Stubbs M and Chaplin DJ (1997) The relationship between extracellular lactate and tumour pH in a murine tumour model of ischaemia-reperfusion. *Br J Cancer* **75**: 319–323

Reinhold HS, van den Berg-Blok AE and van den Berg AP (1991) Dose-effect relationships for glucose-induced tumour acidification and erythrocyte flux. *Eur J Cancer* **27**: 1151–1154

Stamato TD and Jones C (1977) Isolation of a lactic dehydrogenase-A-deficient CHO-K1 mutant by nylon cloth replica plating. *Somatic Cell Growth* **3**: 639–647

Tannock IF (1968) The relation between cell proliferation and the vascular system in a transplanted mouse mammary tumour. *Br J Cancer* **22**: 258–273

Thistlethwaite AJ, Alexander GA, Moylan DJ and Leeper DB (1987) Modification of human tumor pH by elevation of blood glucose. *Int J Radiat Oncol Biol Phys* **13**: 603–610

Varnes ME, Tuttle SW and Biaglow JE (1984) Nitroheterocycle metabolism in mammalian cells. *Biochem Pharmacol* **33**: 1671–1677

Vaupel P, Kallinowsky F and Okunieff P (1989) Blood flow, oxygen and nutrient supply and metabolic microenvironment of human tumors: a review. *Cancer Res* **49**: 6449–6465

Wike-Hooley JL, Haveman J and Reinhold JS (1978) The relevance of tumour pH to the treatment of malignant disease. *Radiother Oncol* **2**: 343–366

© Cancer Research Campaign 1998